# Biology of the non-parasitic phase of the cattle tick *Rhipicephalus* (*Boophilus*) *microplus* in an area of Amazon influence

**DOI:** 10.1186/s13071-024-06220-w

**Published:** 2024-03-14

**Authors:** Daniela P. Sales, Marcelo H. S. Silva-Junior, Caio P. Tavares, Isabella C. Sousa, Dauana M. Sousa, Danilo R. B. Brito, André M. Camargo, Romário Cerqueira Leite, J. L. H. Faccini, Welber D. Z. Lopes, Marcelo B. Labruna, Hermes R. Luz, Livio M. Costa-Junior

**Affiliations:** 1 Post-Graduation Program in Animal Health Defense, State University of Maranhão, Maranhão, Brazil; 2 Laboratory of Parasite Control, Federal University of Maranhão, São Luís, Maranhão Brazil; 3 Federal Institute of Education, Science and Technology of Maranhão, São Luís, Maranhão Brazil; 4 Post‑Graduation Program in Health Sciences, Center of Biological and Health Sciences, Federal University of Maranhão, São Luís, MA Brazil; 5 School of Veterinary and Zootechny of the Universidade Federal de Goiás, Goiânia, Goiás Brazil; 6 Department of Preventive Veterinary Medicine and Animal Health, Faculty of Veterinary Medicine and Animal Science, University of São Paulo, São Paulo, Brazil; 7 Post‑Graduation Program in Northeast Biotechnology Network (RENORBIO), Biodiversity and Conservation, Center of Biological and Health Sciences, Federal University of Maranhão, São Luís, MA Brazil

**Keywords:** *Rhipicephalus (Boophilus)microplus*, Amazon, Temperature, Precipitation, Biology, Brazil

## Abstract

**Background:**

*Rhipicephalus* (*Boophilus*) *microplus* is the most important tick species affecting cattle in the world. Under field conditions, the non-parasitic phase of *R*. (*B*.) *microplus* is unknown in the Amazon biome, including Brazil. The present study aimed to evaluate the non-parasitic phase of *R*. (*B*.) *microplus* in field (grass plots) and laboratory conditions.

**Methods:**

The study was conducted from September 2020 to April 2022 in an Amazonian region (Maranhão State, Brazil). We evaluated the biological parameters of *R.* (*B.*) *microplus* under laboratory and field conditions. Engorged females were exposed to experimental conditions every 14 days, totaling 20 months of study. The following biological parameters were observed: pre-oviposition period, egg mass incubation period, and maximum larval survival period.

**Results:**

Abiotic data (e.g., temperature and humidity) varied little throughout the year. Precipitation was the factor that varied the most throughout the year (dry ~ 30 mm^3^ and rain 400 mm^3^), and the parameters of pre-oviposition and pre-hatching are longer during the rainy season. A possible negative effect of the dry season on the percentage of hatched eggs was observed. Larval longevity in the plots of both control and free females was short (mean ~ 50–60 days), below that recorded for larvae under controlled conditions (mean ~ 95 days).

**Conclusions:**

*Rhipicephalus* (*Boophilus*) *microplus* was able to complete its non-parasitic phase by producing host-seeking larvae in the pasture during all months of the study. The results indicate that *R.* (*B.*) *microplus* can complete up to six generations per year in biome Amazon. To our knowledge, this is the highest number of annual generations for *R.* (*B.*) *microplus* in Latin America.

**Graphical Abstract:**

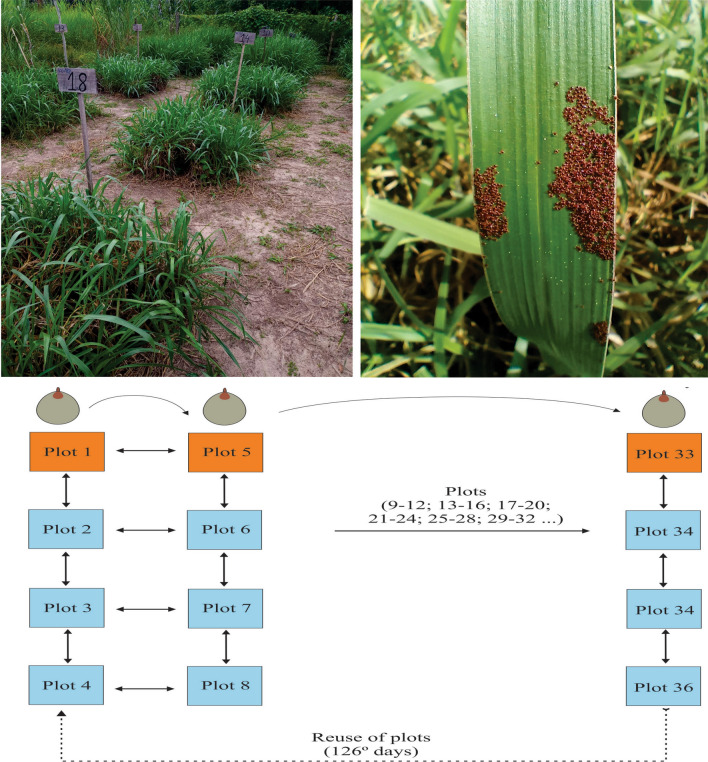

**Supplementary Information:**

The online version contains supplementary material available at 10.1186/s13071-024-06220-w.

## Background

*Rhipicephalus* (*Boophilus*) *microplus* (Canestrini, 1888) is an ectoparasite of cattle that causes damage to livestock ~ US$ 3.2 billion/year in Brazil, such as losses in meat and milk production, devaluation of leather, reproductive disorders, myiasis, anemia, control costs, and loss of animals due to tick fever (caused by *Anaplasma marginale*, *Babesia bovis*, and *Babesia bigemina*) [1–4]. The parasitic phase that comprises the attached larva until the engorged female detachment lasts ~ 21 days [1]. However, the success of its non-parasitic phase depends mainly on abiotic factors. Among these factors, temperature is the main modeler of the non-parasitic phase of *R.* (*B*.) *microplus*, which can shorten this phase under high temperatures or prolong it under low temperatures.

These abiotic factors are essential for the development and survival of *R.* (*B*.) *microplus* in the pasture. For example, the viability of most engorged females and eggs depends on humidity ≥ 70% and temperatures between 20 ℃ and 35 ℃, while the duration of the pre-oviposition and egg incubation periods are inversely proportional to the temperature [1, 5], which explains the distinct generations of *R.* (*B*.) *microplus* in Brazil [1, 6]. Among the abiotic factors mentioned, temperature is the main modeler of the non-parasitic phase of *R.* (*B*.) *microplus*, which can shorten (under high temperatures) or prolong (under low temperatures) this phase. In fact, *R.* (*B*.) *microplus* presents three to five annual generations in Brazil, three in the southern region (temperate climate and average annual temperature of 12–14 ℃) and four to five in the southeast and central west regions (tropical climate and average annual temperature of 20–24 ℃) [1, 6]. In the Amazon biome (North and Northeast regions), under the influence of the equatorial climate and with an average annual temperature of 26–28 ℃, the biology of *R.* (*B*.) *microplus* is still unknown. However, mathematical model-generated data have indicated that in this biome this ectoparasite can reach up to six annual generations [7, 8].

The Amazon region encompasses a vast tropical forest that spans eight countries in South America, and its transformations significantly affect global climate patterns. Over the last half century, the cattle population in the Brazilian Amazon biome has increased tenfold [9]. To promote sustainable intensification and increased productivity, considerable attention has been paid to addressing animal health and welfare. Concomitantly, *R.* (*B*.) *microplus* has also expanded in the Amazon biome, which in addition to parasitizing cattle in this region, can also be found in a variety of wild mammals [10]. Studies indicate that wild hosts can play a secondary role in the life cycle of *R.* (*B*.) *microplus*, maintaining high population densities of this ectoparasite even with adequate anti-tick management [11].

Under the above circumstances, knowledge of *R.* (*B*.) *microplus* biology in the Amazon biome is necessary and important for sustainable cattle farming, in addition to contributing to the understanding of possible impacts of this ectoparasite on wildlife. The objective of this study was to evaluate for the first time the non-parasitic phase of *R.* (*B.*) *microplus* and the climatic factors that interfere in its biology in a region of Amazonian influence in Brazil.

## Methods

### Study site and maintenance of the* Rhipicephalus* (*Boophilus*) *microplus* colony

The study was conducted from September 2020 to April 2022 at the Federal Institute of Maranhão, Campus Maracanã (2°36ʹ59″ S and 44°16ʹ14″ W), located in the municipality of São Luís, Maranhão, Brazil. The area belongs to the Amazon biome, with a medium temperature of 27–29 ℃ and altitude < 100 m. It has a tropical equatorial climate with mean annual precipitation ~ 2900 mm [12, 13]. For the current study, a colony of *R.* (*B.*) *microplus* was obtained from naturally infested cattle in the municipality of Santa Rita Maranhão state (Amazon biome), kept in the Central Animal Facility of the Federal University of Maranhão, Bacanga/São Luís (2°33ʹ09″ S and 44°18ʹ23″ W). To maintain the colony, engorged females of *R.* (*B.*) *microplus* were kept in a biochemical oxygen demand incubator (BOD) at 27 ℃ and relative humidity (RH) > 80% for oviposition. The ticks were fed on two cattle (*Bos taurus taurus*), free from antiparasitic treatments. Each animal received ~ 2000 larvae in the dorsal line per infestation and restrained with a halter to prevent self-cleaning for ~ 2 h after the procedure. During the entire study, cattle received hay and water ad libitum, and 1% of their body weight of commercial pellets with 20% crude protein. For the natural recovery of *R.* (*B.*) *microplus* females at their maximum engorgement, the cattle were kept in stalls with a braided iron floor.

**Figure 1:**
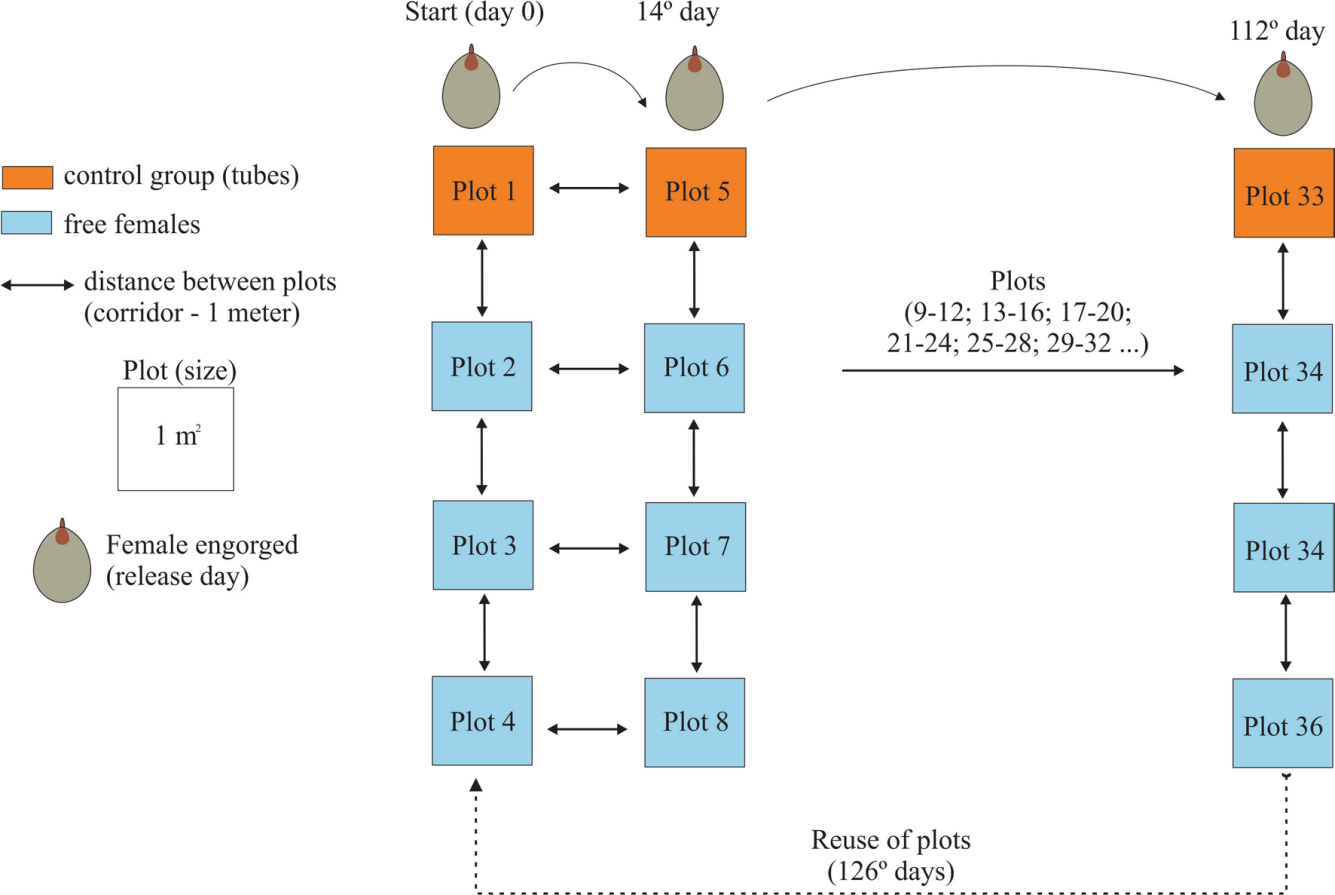
Schematic representation of the study of the non-parasitic phase of *Rhipicephalus* (*Boophilus*) *microplus* in the field. Females were exposed to the field study every 14 days

For the field study, a 200 m^2^ tick-free pasture composed of *Brachiaria brizantha* grass was used, which was divided into 36 plots of 1 m^2^, separated by 1 m and divided by paths free of vegetation. Grass in the plot was left ≈ 30 cm high on the day of tick release (Fig. 1).

### Development of the non-parasitic phase under laboratory and field conditions

We evaluated the biological parameters of *R.* (*B.*) *microplus* under laboratory and field conditions. The following protocol was an adaptation of the described protocols for field studies with engorged females of *Amblyomma sculptum* (= *Amblyomma cajennense *sensu lato) [14] and *Dermacentor nitens* [15] in southeastern Brazil. Between September 2020 and April 2022, every 14 days, 45 engorged females collected from cattle were exposed to experimental conditions the same day and divided into five groups: group (1) five engorged females used as a control group under laboratory conditions placed inside cylindrical tubes in an incubator at 27 ± 2 ℃ and 85 ± 5% RH and full darkness; group (2) ten engorged females used as a control group under field conditions, kept individually inside cylindrical plastic mesh tubes (0.2 mesh, 2 × 5 cm, 18 mm diameter), and placed horizontally on the soil surface under the grass in the first plot, e.g., plot 1, plot 5, and so on (Fig. 1); groups (3, 4, and 5) ten engorged females kept under field conditions and left freely on the soil surface under the grass, protected from sunlight, in the middle of three plots, e.g., plots 2–4 and 6–8 (Fig. 1). When the experiment reached the last plots (33–36), plots 1–4 were reused (already completely free of ticks) (Fig. 1).

The plots of the control groups in the field and in the laboratory were inspected between 8:00 a.m. and 10:00 a.m. every 2 days. The females inside the tubes were inspected to determine the pre-oviposition period (period between release of females and the beginning of oviposition), egg mass incubation period (period between the beginning of oviposition and the beginning of eggs hatching), and maximum larval survival period (period of time from initial hatching to the last day when at least one live larva was observed in the tube). When all larvae had died, the tube contents were discarded on a plate, and the percentage of hatched eggs was visually estimated under a stereoscope microscope [14]. The same procedures were performed with the control females in the incubator.

Plots with free females were also inspected every 2 days for larvae on the grass tips between 8:00 a.m. and 10:00 a.m. The period that elapsed from the release of engorged females into the plot until the first appearance of larvae at the top of the grass was considered the pre-hatch period [16]. Once clusters of larvae were observed at the edges of vegetation, subsequent weeks in which fewer than ~ 10–15 live larvae were present were recorded, and thereafter observations of at least 1 live larva still present in the plot was recorded. When larvae were no longer found by visual inspection of the plot, the grass was swept up to six times with a wooden stick (60 × 7 × 2.5 cm) covered with white flannel. If one or more larvae were caught, they were manually returned to the grass, which was considered to still harbor live larvae. Inspections continued until three sweeps per inspection day failed to detect any larvae for two consecutive weeks, and no larvae could be seen in the vegetation. Larval survival in each plot was determined as the period during which the majority of larvae survived (1–5 live larvae were present in the plot).

### Meteorological data

The region of the current study has only two seasons in the year, popularly called summer (dry season) and winter (rainy season); in the present study the data were grouped into the dry season (July–November) and rainy season (December–June). Climatic variables such as temperature, humidity, and precipitation were obtained from the Agritempo website [17] using the São Luís meteorological station, located 10 km from the area of the present study. These variables were used in an attempt to find any association with the biological parameters obtained in the present study.

### Statistical analyses

The different biological variables of ticks were compared between the 20 months of study in each of the three experimental conditions (control ticks in the incubator, ticks confined in cylindrical tubes in pastures, and ticks released freely in pastures). Development periods were compared using the Student’s *t* test for comparison of two groups and analysis of variance (ANOVA) for more than three groups. The chi-squared test was used to compare hatching rates. The simulation of total generations for *R*. (*B*.) *microplus* was performed by adding the mean pre-hatching period of free females + mean period of the parasitic phase [15]. A significance level of 5% was considered for all reported analyses.

## Results

Every time that engorged females were exposed to field conditions (Fig. 1), corresponding engorged females comprising the control group were exposed to ideal conditions in the laboratory. The mean and standard deviation of the periods of each biological parameter of the non-parasitic phase of *R.* (*B.*) *microplus* under field conditions in the dry and rainy season and in the laboratory are presented in Tables 1 and 2.

**Table 1:** *Rhipicephalus* (*Boophilus*) *microplus* reproductive and survival mean periods for engorged females and unfed larvae, respectively, observed in an incubator (BOD) at 27 ℃ and 85 ± 5% RH and in the field grass plots during the present study

Seasons/biological parameters	Pre-oviposition period (days)	Incubation period (days)	% Egg hatching	Larval maximal survival period (days)
Dry season (plots)	3.75 ± 1.01 a	24.27 ± 2.23 a	77.0 ± 2.01 a	57.78 ± 7.49 a
Rainy season (plots)	4.33 ± 1.23 b	24.33 ± 1.76 a	80.2 ± 0.20 a	56.92 ± 5.85 a
BOD	2.01 ± 0.03 c	25.65 ± 1.27 a	94.11 ± 2.3 a	95.25 ± 5.41 b

Groups of engorged females, confined to cylindrical tubes, were placed inside the incubator and on the soil base under the vegetation of grass at 14-day intervals. The lots of engorged females were formed every 14 days from September 2020 to April 2022; data were grouped for all lots from the same month over the 20-month period; values presented as the mean ± standard deviation; different letters in the same column indicate significantly different mean values (*P* < 0.05)

**Table 2:** Biological data for *Rhipicephalus* (*Boophilus*) *microplus* engorged females released freely on grass plots at day 14

Seasons/biological parameters	Pre-hatching period (days)	Larval maximal survival period (days)
Dry season (plots)	39.58 ± 3.09 a	54.50 ± 4.09 a
Rainy season (plots)	41.43 ± 3.68 b	56.24 ± 5.17 a

The lots of engorged females were formed every 14 days from September 2020 to April 2022; data were grouped for all lots from the same month over the 20-month period; values presented as the mean ± standard deviation; different letters in the same column indicate significantly different mean values (*P* < 0.05)

Females from the control group in the laboratory before oviposition weighed an average of 238 mg. All females in the control group oviposited fertile eggs that presented a hatching rate > 90% (mean 94.11 ± 2.3). When the data were grouped (20 months of study) the females in the incubator took only 2 days to start oviposition (Table 1). The incubation period lasted an average of ~ 26 days (~ 4 weeks) and larvae survived an average for 95 days (~ 13 weeks) (Table 1).

The engorged females used as a control group under field conditions oviposited fertile eggs that resulted in larvae. A total of 23 engorged females died before initiating oviposition, in both dry and rainy seasons. More than half (56.5%) of female losses occurred in the rainy season (13 females). None of the above dead females were replaced during the study. The engorged weight of these females ranged from 190 mg to 400 mg (mean 295 mg). The pre-oviposition period was 3–4 days (mean 3.75 ± 1.01 days) throughout the study. The incubation period had a mean of 24 days in both rainy and dry seasons (Table 1). The hatching rate of the eggs was greater than 70% in most of the study and did not show a significant difference between the dry season (77.0 ± 2.01%) and the rainy season (80.2 ± 0.2%) (*P* = 0.631). However, hatching rates < 50% were observed for 49 females exposed from July to December, especially in the dry season of August (8/20 females) and September (8/20 females) with a hatching rate of 45%.

​Eggs from two females exposed in the rainy season (January and February) showed a hatching rate of 40%. Maximum larval survival in cylindrical tubes in the field was was similar between the dry and rainy seasons (Table 1).

The engorged females kept under field conditions and left freely on the soil surface under the grass had a pre-hatching period longest in the rainy season (Table 2). However, some females had a short pre-hatching period (mean 21 days) when released in April, and females released in June had the longest pre-hatching period (mean 39 days). The larval survival was similar throughout the year (Table 2).

**Figure 2:**
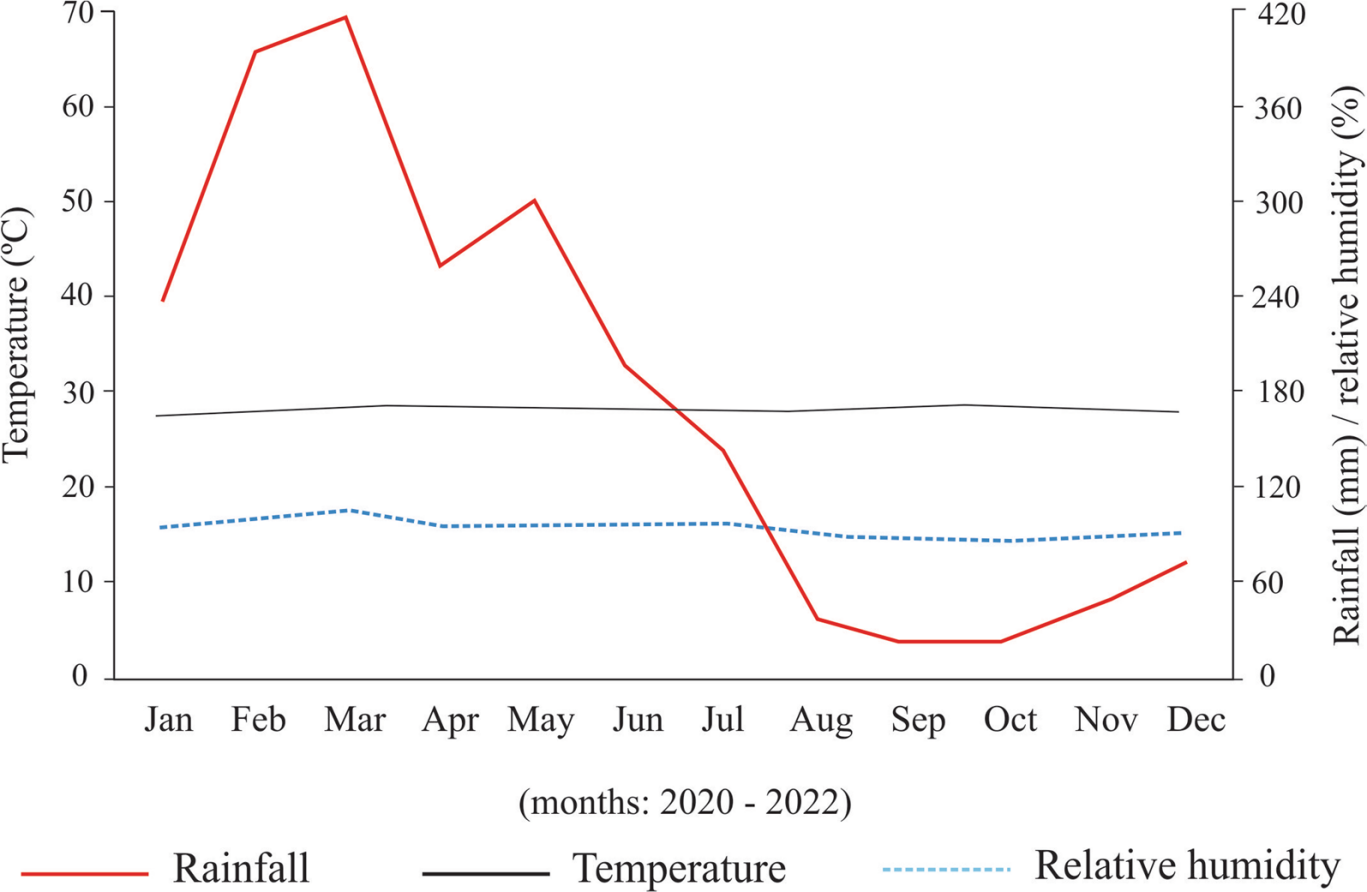
Mean monthly data of temperature (°C), rainfall (mm), and relative humidity (%) of the study site

The climatic data obtained in the present study are shown in Fig. 2. In general, temperature (~ 26–28 ℃) and relative humidity (~ 70–80%) varied little throughout the study, except precipitation, which had high peaks in the hottest months, rainy (February and March) (~ 400 mm).

## Discussion

This is the first field study of the non-parasitic phase of *R.* (*B*.) *microplus* in the Amazon biome. Therefore, the current study is an important contribution for the biology of *R.* (*B.*) *microplus* in this biome, which is the largest in South America.

Temperature and relative humidity are essential in the non-parasitic phase of *R.* (*B.*) *microplus*, often modeling the population dynamics of this Ixodidae in the pasture (e.g., seasonality and density) [1, 5, 18]. During the study of the non-parasitic phase of *R*. (*B*.) *microplus*, the temperature (~ 26–28 ℃) and humidity (~ 70–80%) were the factors that varied less. This explains why there was also little variation in the periods of development of the non-parasitic phase of *R*. (*B*.) *microplus* throughout the year (dry and rainy seasons) in the present study. While the incubation period was similar throughout the year in the plots and incubator (BOD), the pre-oviposition period in the plots (females in tubes) was slightly shorter in the dry season. It is possible that the high temperature and relative humidity > 70% in the dry season (precipitation ~ 30–90 mm) created optimal conditions for the engorged *R*. (*B*.) *microplus* females and accelerated the pre-oviposition period. On the contrary, on days of high rainfall in the rainy season (~ 400 mm) female *R.* (*B.*) *microplus* have interrupted oviposition.

As mentioned previously, the percentage of hatched eggs of *R.* (*B.*) *microplus* depends mainly on relative humidity [1]. In our study, the percentage of hatched eggs varied throughout the year, but not enough to differ statistically between the dry (mean 76.16 ± 4.01 days) and rainy (mean 80. 20 ± 2.03 days) seasons. In fact, the abiotic factors mentioned during the study may have favored hatching values in both dry and rainy seasons. A possible synchronization between rainfall and percentage of hatched eggs was observed between the months of October (end of the dry season) and December (beginning of the rainy season), increasing from an average of ~ 60% to ~ 80%, respectively.

Although the hatching rate of the eggs was high throughout much of the study (> 70%), eggs from 49 females showed hatching rates < 50% in the dry season. The coincidence with the dry season indicates a possible effect of precipitation on the hatching rates of *R* (*B.*) *microplus* eggs, however, not enough data was obtained for us to be able to confirm this hypothesis.

The positive correlation between rainfall and percentage of hatched eggs was reported by de Barros et al. [19]. During the rainy season, the soil is wetter and for longer periods compared with the dry season with only occasional rain; the eggs therefore have greater viability during the rainy season. This hypothesis corroborates other studies for this species of tick, showing the importance of the rainy season in percentage of hatched eggs [1, 19].

Eggs from free females in the plots showed distinct pre-hatching periods, which were slightly shorter in the dry season (mean ~ 39 days) compared with the rainy season (mean ~ 41 days). An expected result, due to the monthly homogeneity of abiotic data (temperature and relative humidity), is found in the present study. Larvae from free females in the plots survived on average ~ 54 days in the dry season and ~ 56 days in the rainy season, similar to the survival time of larvae from engorged females from the control group maintained under field conditions, but below the survival time of larvae and controlled climatic conditions. Temperature may have been a possible determinant for this difference, since at BOD this climatic factor was controlled at 27 ℃, and in most field observations the ambient temperature was ≥ 27 ℃, reaching maximums ~ 40 ℃. In both field and BOD studies the relative humidity was similar, always > 70% RH. Even during the dry season, the relative humidity was greater than 70%, which contributed to the larval survival time being similar to that recorded during the rainy season. In fact, temperature and humidity are the main abiotic factors regulating the larval survival of *R.* (*B.*) *microplus* in the environment [1, 19, 20].

In practice in the Amazon biome, cattle are exposed to *R.* (*B.*) *microplus* larvae throughout the year. Considering that the current study started on 9 September 2020 (day of release of the engorged females), we can infer that these females (first peak/September 2020) could produce larvae in search of a host in the pasture 5.7 weeks later according to the mean pre-hatching period for September. Adding a parasitic phase of 3.1 weeks on cattle susceptible to *R.* (*B.*) *microplus*, the interval of the first (September 2020) and second (November/December 2020) peak of engorged females on cattle can be supported by the following numbers [5.7 + 3.1 = 8.8 weeks (~ 62 days)]. Therefore, detached engorged females from the second peak (November/December 2020) may have produced larvae in search of hosts in the pasture 5.9 weeks and 6.0 weeks later, which added to a parasitic phase of 3.1 weeks, giving an interval of 62–63 days between the second (November/December 2020) and the third (January/February 2021) peaks of engorged females on cattle. The engorged females of the third peak may have produced larvae searching for a host in the pasture 6.0 weeks later, added to the parasitic phase of 3.1 weeks, giving an interval of 64 days between the third (January/February 2021) and the fourth (March/April 2021) peaks of engorged females in the pasture. Likewise, engorged females from the fourth peak may have produced host-seeking larvae in the pasture 6.1/6.0 weeks later, added to the parasitic phase of 3.1 weeks, giving an interval of 64 days between the fourth peak (March/April 2021) and the fifth peak (May/June 2021) of engorged females in pasture. Finally, detached engorged females from the fifth peak (May/June 2021) may have produced host-seeking larvae in the pasture 6.0 weeks and 6.6 weeks later, which added up to a parasitic phase of 3.1 weeks, giving an interval of 64–68 days between the fifth (May/June 2021) and sixth (July/August) peaks of engorged females on cattle. In this context, it is possible to conjecture that in the Amazon biome, *R.* (*B.*) *microplus* can complete up to six generations per year in a perfect scenario of optimal abiotic factors (high temperature and relative humidity) and constant presence of susceptible hosts. This hypothesis is supported by the models reported by Evans [7] and Hernández-A et al. [8] determining ≥ 5.0 generations per year for *R.* (*B.*) *microplus* in the Amazon biome.

In Brazil, *R.* (*B.*) *microplus* is known to have three generations per year in the Southern region and 4–5 generations per year in the Cerrado biome in the Southeastern and Central-Western regions of the country [1, 6, 21, 22]. This difference in the number of generations between the Southern, Southeastern, and Central-Western regions can be associated with different climatic factors (temperature, humidity, and precipitation) throughout the country. Temperature is an important factor since its increase shortens the non-parasitic phase of *R.* (*B.*) *microplus* and consequently increases the number of annual generations [1]. In fact, the number of annual generations of *R.* (*B.*) *microplus* mentioned above increased in the south–north direction of Brazil, indicating that under high and constant temperatures throughout the year, high relative humidity, this ixodid tends to shorten its non-parasitic phase. Therefore, the possibility of *R.* (*B.*) *microplus* reaching up to six annual generations recorded in the present study is surprising, but expected.

## Conclusions

The results of the present study show that female *R.* (*B.*) *microplus* in the Amazon biome can produce viable larvae in all months of the year. Due to the high temperatures and relative humidity throughout the year, the non-parasitic phase of this ixodid was accelerated and could complete up to six generations per year. Variation in rainfall may be an important climatic factor in the population dynamics of *R.* (*B.*) *microplus*, with a possible deleterious effect in months of excess rainfall (> 400 mm^3^), mainly regarding its development periods. The data presented here can be useful to understanding the life cycle of *R.* (*B.*) *microplus* in the Amazon biome, as well as serving as the basis for other investigations within this biome that covers a large part of South America. Other field studies are necessary to create a control program for this ixodid in the study region, for example, to quantify parasitic intensity in cattle and the environment.

### Supplementary Information

**Additional file 1: Table S1.** Original data of the biology of *R. microplus* in the present study.

## Data Availability

All data supporting the findings of this study are available within the paper and its associated file.
